# Supplementary figures and images for: The Complex Transcriptional Landscape of Magnetosome Gene Clusters in Magnetospirillum gryphiswaldense
Source: mSystems. 2021 Sep 14;6(5):e00893-21. doi: 10.1128/mSystems.00893-21 (PMC8547445; doi:10.1128/mSystems.00893-21)

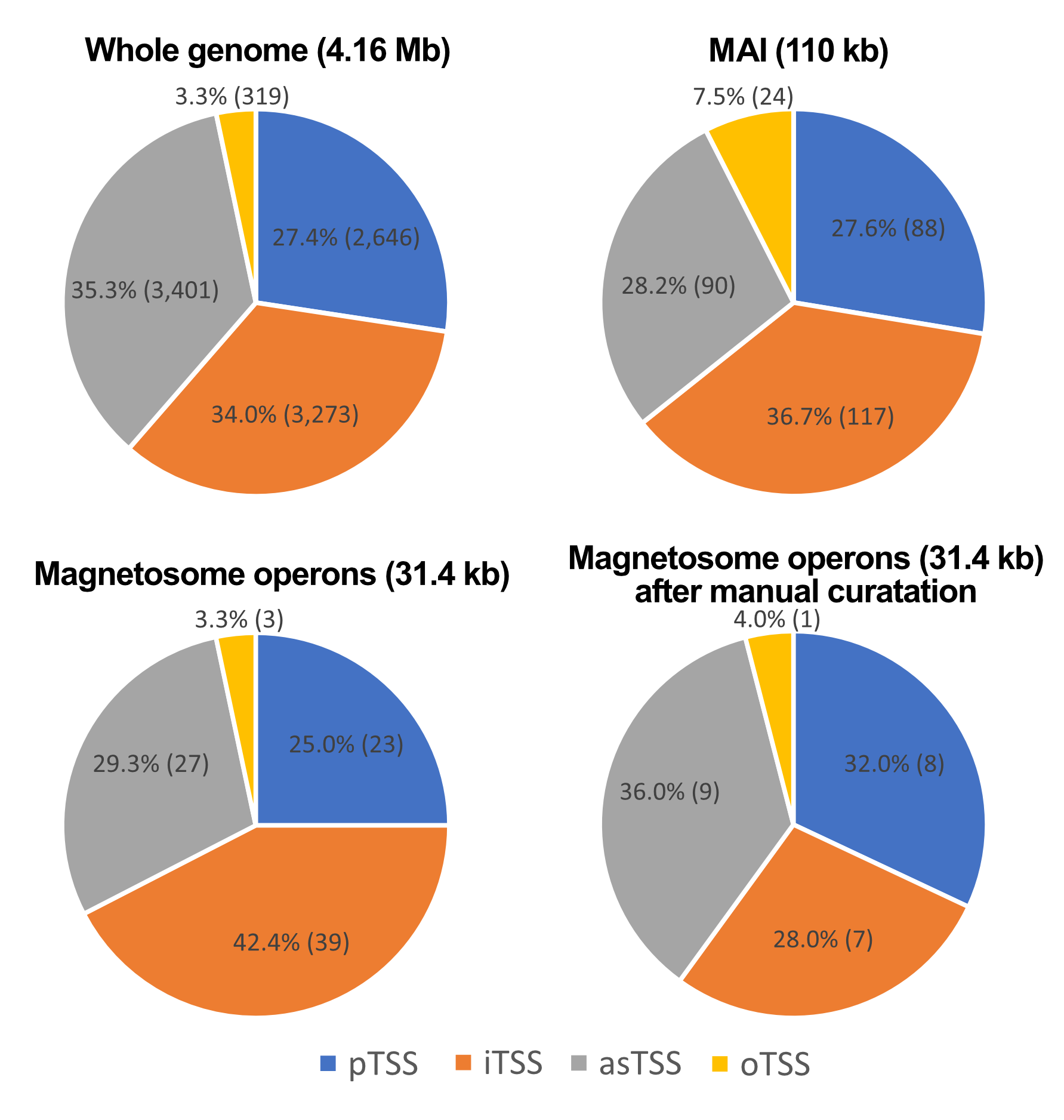

Supplement: FIG S1 [file msystems.00893-21-sf001.tif]

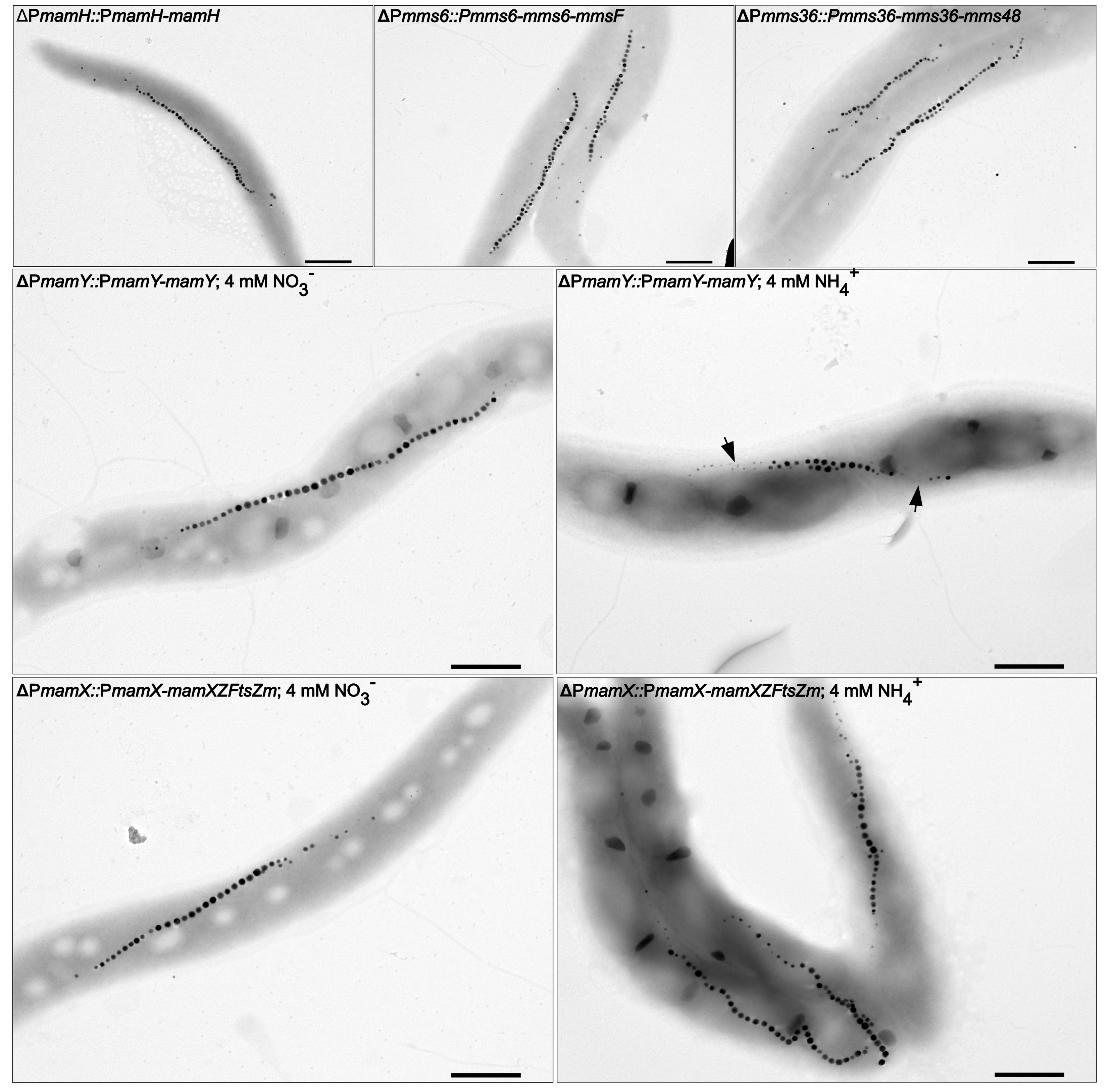

Supplement: FIG S2 [file msystems.00893-21-sf002.tif]

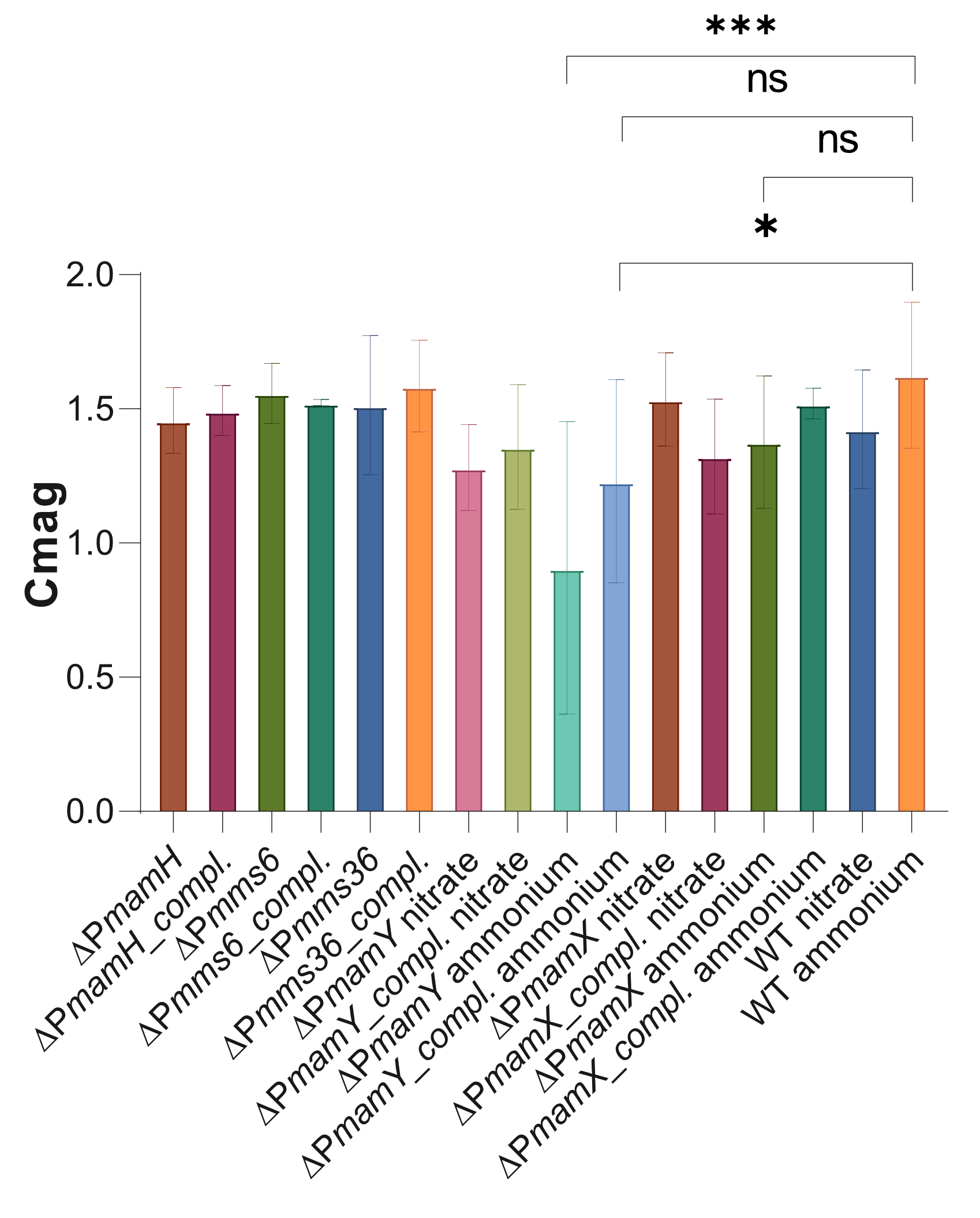

Supplement: FIG S3 [file msystems.00893-21-sf003.tif]

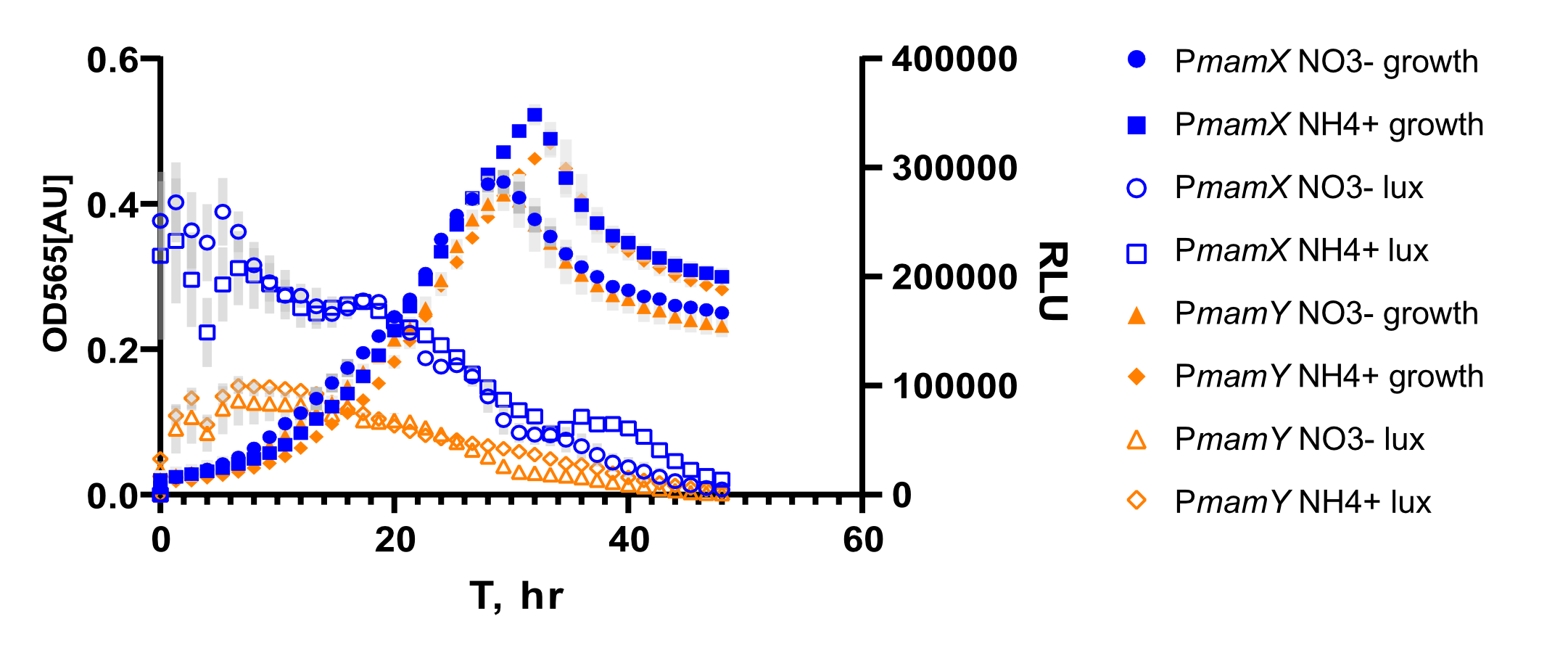

Supplement: FIG S4 [file msystems.00893-21-sf004.tif]
