# Supplementary material for: The Complex Transcriptional Landscape of Magnetosome Gene Clusters in Magnetospirillum gryphiswaldense
Source: mSystems. 2021 Sep 14;6(5):e00893-21. doi: 10.1128/mSystems.00893-21 (PMC8547445; doi:10.1128/mSystems.00893-21)
Supplement: TABLE S2 [file msystems.00893-21-st002.pdf]

Table S2: The full set of TSS found in the major MagOPs. The TSS supported by both Cappable-seq and WTSS were selected for bioluminescence analysis by manual curation.

TSS start	Strand	Classification	RRS	Capseq_score	Selected for the bioluminescence analysis after manual curation (TSS No./Putative promoter)
feoAB1op					
269852	Fwd	pTSS	112	2.5	1/P_{feoA1}
269894	Fwd	pTSS	19	1.5	
270478	Fwd	iTSS	129	4.3	
270748	Fwd	iTSS	18	1.5	
271180	Rev	asTSS	27	2.0	
mms6op					
307433	Rev	pTSS	1270	3.1	2/P_{mms36}
307467	Rev	oTSS	112	1.5	
307629	Rev	iTSS	575	2.9	
308388	Rev	pTSS	54	3.0	
308415	Fwd	asTSS	15	1.2	
308433	Rev	pTSS	20	1.6	
308678	Rev	oTSS	188	2.8	3/P_{mms6}
308808	Rev	oTSS	41	1.1	
mamGFDCop					
308983	Fwd	pTSS	27	2.0	4/P_{mamG}
309202	Fwd	iTSS	31	2.2	
309668	Rev	asTSS	39	2.6	
309820	Rev	asTSS	28	2.1	
310191	Fwd	iTSS	24	1.9	
mamABop					
321356	Fwd	pTSS	269	1.1	5/P_{mamH}
321463	Fwd	iTSS	36	2.5	
322571	Rev	asTSS	190	1.9	
322593	Fwd	iTSS	11352	1.4	6/$P_{(mamH)}$
323000	Fwd	iTSS	19	1.5	
323482	Fwd	iTSS	24	1.9	7/$P_{(mamE)}$
323846	Rev	asTSS	39	1.6	
325254	Fwd	iTSS	256	1.0	
326474	Fwd	iTSS	318	1.2	
326530	Fwd	pTSS	18	1.5	
327029	Rev	asTSS	15	1.2	
327354	Rev	asTSS	20	1.6	
327686	Fwd	iTSS	303	2.5	8/$P_{(mamL1)}$
327876	Fwd	iTSS	94	1.0	9/$P_{(mamL2)}$
327962	Fwd	iTSS	18	1.5	
328474	Rev	asTSS	19	1.5	
328970	Fwd	iTSS	15	1.2	
329292	Fwd	iTSS	21	1.7	
329329	Rev	asTSS	85	3.7	
329434	Fwd	iTSS	59	1.6	
329767	Fwd	iTSS	293	1.2	

329802	Fwd	iTSS	17	1.4	
330355	Rev	asTSS	23	1.8	
330492	Rev	asTSS	16	1.3	
330974	Rev	asTSS	30	2.2	
331477	Rev	asTSS	17	1.4	
331685	Fwd	iTSS	24	1.9	
332122	Fwd	iTSS	1309	1.5	10/$P_{(mamO)}$
332212	Fwd	iTSS	20	1.6	
333007	Fwd	pTSS	73	2.5	11/$P_{(mamP)}$
333061	Rev	asTSS	69	3.4	
333093	Fwd	iTSS	100	3.9	12/$P_{(mamA)}$
333317	Fwd	iTSS	27	2.0	
333526	Fwd	iTSS	94	3.8	
333786	Rev	asTSS	63	3.3	
333959	Fwd	iTSS	92	3.8	
334067	Fwd	iTSS	85	1.7	
334074	Rev	asTSS	22	1.7	
334094	Rev	asTSS	18	1.5	
334435	Fwd	iTSS	682	1.7	13/$P_{(mamQ)}$
335193	Rev	asTSS	33	2.3	
336749	Rev	asTSS	30	2.2	
336981	Fwd	pTSS	32	1.3	
337051	Fwd	iTSS	66	3.3	
mamXYop					
366353	Rev	iTSS	19	1.5	
366668	Fwd	asTSS	17	1.4	
366991	Rev	iTSS	28	2.1	
367249	Rev	iTSS	59	3.2	
367495	Fwd	asTSS	22	1.7	
367833	Rev	iTSS	21	1.7	
368342	Fwd	asTSS	17	1.4	
368801	Rev	iTSS	39	2.6	
369110	Rev	iTSS	144	4.5	
369133	Fwd	asTSS	243	4.2	
369729	Rev	pTSS	315	2.1	14/P_{mamX}
370214	Fwd	asTSS	120	1.9	
370996	Rev	pTSS	15	1.2	15/P_{mamY}
371007	Rev	pTSS	368	3.0	16/P_{mamY}
371052	Rev	pTSS	586	2.6	
