# Supplementary material for: The Complex Transcriptional Landscape of Magnetosome Gene Clusters in Magnetospirillum gryphiswaldense
Source: mSystems. 2021 Sep 14;6(5):e00893-21. doi: 10.1128/mSystems.00893-21 (PMC8547445; doi:10.1128/mSystems.00893-21)
Supplement: TABLE S3 [file msystems.00893-21-st003.pdf]

Table S3 Characteristics of the promoters tested in this study. For each promoter, RLUmax values measured with optRBS ans natRBS are shown in three representative clones.

Promoter	Fragment sequence 5'-3'	Fragment length, bp	Position to predicted TSS	Promoter type by position in the operon	Activity, maximal emmitted light (RLUmax), with native RBS			Activity, maximal emmitted light (RLUmax), with optRBS		
					Clone 1	Clone 2	Clone 3	Clone 1	Clone 2	Clone 3
P*mamH**	GGCATCCTGATCGGTAGGCGATGGCGCAAAGATGTGACGTC<br>TTGTTTCGAGCAGATGCGCGAAATGTGCTAGCGCGCTAAGT<br>GTGCCACATTGCGAGGTTCCTTCGTATGAACCCAGTTTTTAT<br>GGCTTGTCAACCGACCTCGATTCGTTGCTATAGTCCGTGCGA<br>ATTGGAGGTGAATTGTGACGGGA	189	+165 -23	primary	23,270.05 ± 9,077.89	10,467.74 ± 1,684.37	13,395.54 ± 3,325.41	ND	ND	ND
P*(mamH)*	GTTCAGATTGGTGGGTTTTTATTTGATTACATTGGCCCTCCTG<br>CGCCTTTTATCTTTACGGGCGTTGGAAATTTGATTATATCAGC<br>GTATGCGTTGCGTCTTGTGAAAAGTGAGGCGCGTGGGGTTG<br>GCGGGGGTAACACCCCAGGGGATGACGAAGTGGCCTAGCG<br>CCGCTTGTGTTCTGTATCAAGACTGGAGAACGTTT	202	+89 -112	intragenic	215,346.90 ± 10,064.55	149,722.90 ± 28,683.55	117,963.00 ± 12,001.70	177,739.8 ± 3,106.58	475,593.90 ±98,326.13	112,870.50 ±5,441.33
P(*mamE*)	GTTTCGCCAATCCCACCACTCGGTCGGTGGAGAATATCGGC<br>ACCGGCGTTATCGTCCGCAATGACGGCTTCATCGTCACCAA<br>TTACCATGTGGTGCGCGGGGCCAATTCGGTGTACGTCACCG<br>TAAAGGACGAGGAGATCAG	142	+97 -44	intragenic	6,620.39 ± 1,565.49	7,712.31 ± 3,187.51	4,920.84 ± 677.12	10,114.83 ± 314.56	9,124.04 ± 1,787.45	7,703.09 ± 225,05
P*(mamL1)*	ATGGGGGATGTCATTTTGGTTGGCGAAGATGCGTGGCTGTC<br>CAGGACATTGGTATGGTAAGAGTGATCGGATCGTTGGTGTTC<br>GGCGGCTTGATCCTGCTGCTGGCATCGTCCAACGCC	119	+70 -48	intragenic	ND	ND	ND	22,164.34 ± 3,965.70	11,467.95 ± 408.12	14,942.13 ± 1,032.91
P*(mamL2)*	TCGTTGGTGTTCGGCGGCTTGATCCTGCTGCTGGCATCGTC<br>CAACGCCCATATGGTGGAAACCCGATTTGGGCCACTGATAA<br>TGCTTGCGCCACATTTCGTGGTTCTTGGCATTACGTTTTTTCT<br>TGGTTTCGCCATTGGGATTGTGTTGGTGTTTGCGAATGTTAT<br>GAAACGGCGCAAGCATA	184	+161 -22	intragenic	6,290.25 ± 355.17	5,535.89 ± 83.24	6,144.78 ± 157.91	ND	ND	ND
P*(mamL1)*+P*(mamL2)***	ATGGGGGATGTCATTTTGGTTGGCGAAGATGCGTGGCTGTC<br>CAGGACATTGGTATGGTAAGAGTGATCGGATCGTTGGTGTTC<br>GGCGGCTTGATCCTGCTGCTGGCATCGTCCAACGCCCATAT<br>GGTGGAAACCCGATTTGGGCCACTGATAATGCTTGCGCCAC<br>ATTTCGTGGTTCTTGGCATTACGTTTTTTCTTGGTTTCGCCAT<br>TGGGATTGTGTTGGTGTTTGCGAATGTTATGAAACGGCGCAA<br>GCATAAACTGCCTGGGAAAAACATCGTCATTAAGCGCTGACA<br>TTCCATGCTCCGTCGGAGCTGGGCCGGGGCTTTGGAGTAGG<br>TAAGG	338	+70 -267; +232 -105	intragenic	12,479.97 ± 1,469.04	13,228.65 ± 251.33	14,061.37 ± 419.79	ND	ND	ND
P*(mamO)*	CGGATCAGTCTGCAGAACGCCATTGCCAACAGTTCGGTCCT<br>GGTGTTCTGGGCCTCGGTCGCCGGTTCAGTGGTAGCCTTTA<br>TTCATGGCGGCAGCACCGGTCTTATTCATTGGGAGGCTCCG<br>GTAACATTGGCGCTGGTGATGATCCCAGGTGCTTATGTTGGC<br>GGCATTCTCGGCGCTCGGTTGATGCGGGTGTTGCCTGTCCG<br>GGTGCTTAAGGGTATCTATGCGGCAACCATGGCCGCTATCG<br>CCATCAAGATGCTGACAACGGTGTGATGGATATGGTTTTGGG<br>ATTAGCCGGGA	300	+231 -68	intragenic	2,156.629 ± 1,099.40	2,735.47 ± 1,751.12	2,469.75 ± 473.44	55,643.88 ± 1,455.33	39,911.96 ± 5,745.28	35,344.63 ± 3,490.30
P*(mamP)*	GCACCCCGGTCTTGCGCAAACTGACCGTGGTTTTGCGCGCG<br>GCCGAAGGCGGTTTGGGCTTTGCCCAGTTGGAAGGCGCCC<br>CCATGATCCTTCCGGGCGATCCTCGGCCCCATGGATATCGC<br>GGCGCCTGCACCGATTGCCATCCGGTAGGCCAGGGATTCG<br>AATTGTCGCCCGATCCTGATCTCATCAGCCTGCCGCCTTCG<br>GCCATAACGCGTGATGCGGTTACCCGGGGCGTCAGTCCCCA<br>CGAAGTTCGTGGCCCGTGCGAAGCTTGCCACGTGATAAATT<br>AGCCAGAGCCACCGGATTGGGATTT	310	+290 -19	intragenic	15,400.23 ± 584.39	16,425.34 ± 736.31	15,700.55 ± 413.48	27,361.36 ± 1,422.60	23,292.31 ± 2,802.12	26,600.00 ± 2,286.92
P*(mamA)*	GTGATAAATTAGCCAGAGCCACCGGATTGGGATTTTGGAGA<br>ACAGTATGTCTAGCAAGCCGTCGAATATGCTTGATGAAGTTA<br>CCCTGTATACTCACTACGGCCTATCCGTGGCGAAGAAGCTC<br>GGTGCGAATAGGAGATCAG	143	+101 -41	intragenic	ND	ND	ND	6,448.73 ± 286.87	6,874.21 ± 716.73	5,741.43 ± 297.54
P*(mamQ)*	ATGATGACCTCGCTGGTGGATATGGAAGACCGGATCGCCAG<br>CAAGAGGGAGGAATTTAACGCATCTGCCGCGACCTATAATG<br>TGGCAATCACCAAGTGGCCGTGGGATTATCTGGCAATGATC<br>ACCGGGTTCAAGCGCGTGGAGTATTTCCACGAAAAGCCTGC<br>AGGCGACACGCCGATCATT	183	+118 -64	intragenic	20,086.64 ± 1,335.57	16,833.18 ± 868.32	19,678.62 ± 218.76	38,493.87 ± 7,446.63	39,468.19 ± 890.16	45,952.15 ± 2,931.38

P*mamG**	CAACTTTTTCGCTTTACTAGCTCTTAGTTCTCCAATAAATTCC<br>CTGCGTCGATTTTAAGGGGCAGAGAGGGAATCGTGCATACA<br>CTTGCCGGAGATCAG	99	+48 -50	primary	325,856.80 ± 13,377.86	184,087,10 ± 95,993.28	131,925.3 ± 40,384.65	ND	ND	ND
P*mms6**	ACCTGTCAATTCCTTCCCTGGTCATCGCTGCGCGCCATGCCT<br>GCCAAACAGGGTGCTTTGCTCACGGTGGCAGATTTCTGTGG<br>GGAGGTTTAAGGTGGTGGTGGTCATTGGCGTTAAGGGATCG<br>TCCCGTGCTTACTCTTTGGGATTTGTCGACAATTATTGGGCTT<br>GTGGTTTTGGCGGGAGCTGCGGTGCTTCGCTGTGTCCACAA<br>GAACCGCGACAGCGGGGCCGGGTGCAACAGGGGCTGCTGT<br>CAAAACCCGCGCAGAGGCAAATGCGAATAACCCGCAGCTAG<br>CGGGGGCGGTGCAGATAATAATCGCTCGTAACTTCCATGTA<br>ATAGTGTTTTTAGCCCCATACAGGTTGTCCTATTCGGCAAC<br>CTCATGGTATTGAATTGTATGTCTGGTGAGGCTGATGTTTTTC<br>AGTCCCGCACTACTTGATTGCTAAGGAGAACTCTG	450	+394 -55	primary	66,616.58 ± 5,149.73	35,956.18 ± 1,323.88	41,808.58 ± 1,777.31	ND	ND	ND
P*mms36*	GGTTCGGCCTCGGGTGATTTACGTCTGATGCCCGGCTGTGC<br>AGGGCATCAGCTGTTTCAGGACCGTTTCATCTATAATATTAAT<br>GTCTTGCCAAAGGGATCGGTGATCGTGAAAATCGCCGTTGC<br>GGGGCGGTTGGGCCCGGTTGCAGGCCGCGCAGCTAGCGGA<br>AGCGGAGCCA	175	+124 -50	primary, within operon	29,347.87 ± 2,524.35	36,089.24 ± 2,088.05	34,664.94 ± 5,600.79	ND	ND	ND
P*mamY*	GGCAGCCTCATTTAAACATTCAGGACGCGCTGCCATATTATA<br>CTATTCCGAAATTAATACTTAGCACCACTTTCCCAACGGACT<br>CTTAGGCAATGCAGGTCCTCACAAACAAAAGGCGGTGTTGC<br>GATTAATGCGCATCTTCCGGTATTGACGGACTCGCCACAAAG<br>CCATATCTATCCCTTGTGTCCAAGCTACCTTGCCGGTCAGCT<br>TGCTGTTACTGTGGCAGTATCCTATACACACCAGAACATTTT<br>GGCGACCACCACAGCCACCACGGGAGCAGCCCTT	285	+228 -56	primary	69,670.43 ± 7,322.48	88,076.17 ± 6,500.96	75,163.23 ± 18,715.86	ND	ND	ND
P*mamX*	CGAAAGCCGACGTCCGAAATTGCATGGCCGCCATTTCCAGC<br>TACGGTCCAGACGGAAGGCCGATGGTCATTCCATTGCGTAT<br>GGGAATATGAGATACTTCGGCACTTTCATCGCTAGCGCATCG<br>GGAATCGGTAGCTCCACTTTGACCACAGTCGAACTCCCGTG<br>AACACCAAAGCCGTTGCACATCCCGACATCGCCGTCTGGAT<br>C	207	+104 -102	primary, within operon	2,886.03 ± 567,15	2,603.45 ± 620,14	2,606.91 ± 1,105.23	174,463.30 ± 9,932.38	337,600.00 ± 14,245	243,683.9 ± 16,751.86
P*feoAB1*	GTGCCGAGGGAAAACATCTGCTTGCTCCCATCGCAAGTTGG<br>CTGAATGACCTTTGTGATGCGACTTGCCGGGTGGCCCAACA<br>TTGGATTTTGCGCCTTGACTCGGCAGCAAGATTGAGGGAAG<br>ATGCGCTGAATTAATCGCAACTCATTCGCAATTGCTTTCGATT<br>ATTGCGGCATTAACAAATAAGGAGGAGTTCCAGG	200	+130 -69	primary	143,000.80 ± 3,876.68	100,592.90 ± 12,752.28	118,859.00 ± 4,329.75	ND	ND	ND
P-less Control	GAAGAAGCTCGGTGCGACTAGGAGATCAG	29	-	-	ND	ND	ND	2,648.16 ± 465,76	2,141.53 ± 162,66	3,213.64 ± 496,32

* previously reported promoter containing regions (Schübbe et al., 2006)

** Comprises the putative promoters corresponding to both TSS found within gene mamL, P(mamL1) and P(mamL2)
