# Supplementary material for: The Complex Transcriptional Landscape of Magnetosome Gene Clusters in Magnetospirillum gryphiswaldense
Source: mSystems. 2021 Sep 14;6(5):e00893-21. doi: 10.1128/mSystems.00893-21 (PMC8547445; doi:10.1128/mSystems.00893-21)
Supplement: TABLE S4 [file msystems.00893-21-st004.pdf]

Table S4 Native RBS predicted within MagOPs that were used for the promoter evaluation

RBS sequence	String	Start position	Distance to start codon, bp	Protein*
AGGAGA	-	308,344	6	Mms6
CGGAAG	-	307,396	8	Mms36
CGGAGA	+	308,979	4	MamG
TGGAGG	+	321,361	6	MamH
TGGAGA	+	322,694	6	MamI
TGGAGC	+	325,237	6	MamJ
TGGAGT	+	327,940	7	MamM
TGGAGA	+	333,027	5	MamA
AGGAAT	+	334,544	9	MamR
GGGAGC	-	370,963	7	MamY
AGGAGGAG	+	269,907	7	FeoA1

* Indicates the protein which translation is controlled by an RBS
