# Supplementary material for: The Complex Transcriptional Landscape of Magnetosome Gene Clusters in Magnetospirillum gryphiswaldense
Source: mSystems. 2021 Sep 14;6(5):e00893-21. doi: 10.1128/mSystems.00893-21 (PMC8547445; doi:10.1128/mSystems.00893-21)
Supplement: TABLE S1 [file msystems.00893-21-st001.pdf]

Table S1 Oligonucleotides used in this work. The restriction enzymes are underlined, optRBS is highlighted in red.

Primer name	Sequence 5' - 3'	Restriction enzyme
	Bioluminescence reporter experiment	
XhoI-PfeoAB1 fw	GGGCTCGAGGTGCCGAGGGAAAACATCTG	XhoI
NdeI-PfeoAB1 rev	CCCCATATGCCTGGAACTCCTCCTTATTTGTTAATG	NdeI
XhoI-Pmms6 fw	GGGCTCGAGACCTGTCAATTCCTTCCCTG	XhoI
NdeI-Pmms6 rev	GGGCATATGCAGAGTTCTCCTTAGCAATC	NdeI
XhoI-Pmms36 fw	GGGCTCGAGGGTTCGGCCTCGGGTGATTTACG	XhoI
NdeI-Pmms36 rev	CCCCATATGTGGCTCCGCTTCCGCTAGCTGCGCGG	NdeI
XhoI-PmamG fw	GGACTCGAGCAACTTTTTCGCTTTACTAG	XhoI
NdeI-PmamG rev	CCCCATATGCTGATCTCCGGCAAGTG	NdeI
XhoI-PmamH fw	AAACTCGAGGGCATCCTGATCGGTAGGCG	XhoI
NdeI-PmamH rev	GGGCATATGTCCCGTCACAATTCACCTCC	NdeI
XhoI-P(mamH) fw	GGGCTCGAGGTTCAGATTGGTGGGTTTTTATTTG	NdeI
NdeI-optRBS-P(mamH) rev	GGGCATATGCTGATCTCCTAAACGTTCTCCAGTCTTG	XhoI
NdeI-natRBS-P(mamH) rev	CCCCATATGAAACGTTCTCCAGTCTTGATACAG	NdeI
XhoI-P(mamE) for	GGGCTCGAGGTTTCGCCAATCCCACCACTC	NdeI
NdeI-optRBS-P(mamE) rev	CCCCATATGCTGATCTCCTCGTCCTTTACGGTGACGTAC	NdeI
NdeI-natRBS-P(mamE) rev	CCCCATATGTATCCCGCTCCACCCTCAAAG	NdeI
XhoI-P(mamL1) for	GGGCTCGAGATGGGGGATGTCATTTTGGTTG	XhoI
NdeI-optRBS-P(mamL1) rev	GTTCATATGCTGATCTCCTGGCGTTGGACGATGCC	NdeI
P(mamL2) fw	TCGTTGGTGTTCGGCGGCTTGAT	-
optRBS-P(mamL2) rev	CTGATCTCCTTATGCTTGCGCCGTTTCATAAC	-
NdeI-natRBS-P(mamL2) rev	CCCCATATGCCTTACCTACTCCAAAGCC	NdeI
XhoI-P(mamO) fw	GGGCTCGAGCGGATCAGTCTGCAGAACGCC	XhoI
NdeI-optRBS-P(mamO) rev	CCCCATATGCTGATCTCCTTCCCGGCTAATCCCAAAACC	NdeI
NdeI-natRBS-P(mamO) rev	CCCCATATGTCCCGGCTAATCCCAAAACC	NdeI
XhoI-P(mamP) fw	GTTCTCGAGGCACCCCGGTCTTGCGC	XhoI
NdeI-optRBS-P(mamP) rev	CCCCATATGCTGATCTCCTAAATCCCAATCCGGTGGCTC	NdeI
NdeI-natRBS-P(mamP) rev	CCCCATATGACTGTTCTCCAAAATCCCAATCCG	NdeI
XhoI-P(mamA) fw	GGGCTCGAGGTGATAAATTAGCCAGAGCCACC	XhoI
NdeI-optRBS-P(mamA) rev	CCCCATATGCTGATCTCCTATTCGCACCGAGCTTCTTC	NdeI
XhoI-P(mamQ) fw	GTGCTCGAGATGATGACCTCGCTGGTGGATATG	XhoI
NdeI-optRBS-P(mamQ) rev	CCCCATATGCTGATCTCCTAATGATCGGCGTGTCGC	NdeI
NdeI-natRBS-P(mamQ) rev	CCCCATATGTGCTGTCCAAATCAATTCTTGGATTCC	NdeI
XhoI-PmamY fw	GGGCTCGAGGGCAGCCTCATTTAAAC	XhoI
NdeI-PmamY rev	CCCCATATGAAGGGCTGCTCCCGTGGTGGCTGTGG	NdeI
XhoI-PmamX fw	GGGCTCGAGCGAAAGCCGACGTCCGAAATTG	XhoI
NdeI-optRBS2-PmamX rev	CCCCATATGCTGATCTCCTGATCCAGACGGCGATGTC	NdeI
NdeI-natRBS-PmamX rev	CCCCATATGGATCCAGACGGCGATGTCGGGATGTG	NdeI
Control + string	GGGCTCGAGGAAGAAGCTCGGTGCGACTAGGAGATCAGCATATGGGG	XhoI, NdeI
Control - string	CCCCATATGCTGATCTCCTAGTCGCACCGAGCTTCTTCCTCGAGCCC	XhoI, NdeI
	Promoter sequence substitution	
Pmms6_LHR fw	GCGATGGCCAGATACAGAACCAGGACCTG	-

Pmms6_LHR_PFS rev	GAATGGATGCGAGTAATGGTGCCTGCTCAGATCGCC	-
Pmms6_LHR_PFS fw	GGCGATCTGAGCAGGCACCATTACTCGCATCCATTC	-
Pmms6_PFS_RHR rev	TAGTGTTTTTTAGCCCCATCGATTGAGGACCTTCAGTG	-
Pmms6_PFS_RHR fw	CACTGAAGGTCCTCAATCGATGGGGCTAAAAAACACTA	-
Pmms6_RHR rev	GATGCAGCGCGAACATGGCCAGGATG	-
Pmms36_LHR fw	GCGACATGAAAATCAACAGGACGATCAGGCGT	-
Pmms36_LHR-PFS rev	GAATGGATGCGAGTAATGGGAAGCGGAGCCAATGG	-
Pmms36_LHR-PFS fw	CCATTGGCTCCGCTTCCCATTACTCGCATCCATTC	-
Pmms36_PFS-RHR rev	CATCAGCTGTTTCAGGACCGATTGAGGACCTTCAGTGC	-
Pmms36_PFS-RHR fw	GCACTGAAGGTCCTCAATCGGTCCTGAAACAGCTGATG	-
Pmms36_RHR rev	CTTCGCTGTGTCCACAAGAACCGCGAC	-
PmamH_LHR fw	CCTTCAATTACGGTTTCCACGGTCTCGGC	-
PmamH_LHR_PFS rev	GAGAATGGATGCGAGTAATGTTAGCGCGCTAGCACATTTC	-
PmamH_LHR_PFS fw	GAAATGTGCTAGCGCGCTAACATTACTCGCATCCATTCTC	-
PmamH_PFS_RHR rev	CAGGTTCCATTCCCGTCACCGATTGAGGAGCTTGAGTG	-
PmamH_PFS_RHR fw	CACTCAAGCTCCTCAATCGGTGACGGGAATGGAACCTG	-
PmamH_RHR rev	GCCGAGAACAGAACCGAGCAAGTCTTTTGGTGC	-
PmamY_LHR fw	CAAGGCCCACAACTCGCCATCCTCGAAG	-
PmamY_LHR-PFS rev	GAGAATGGATGCGAGTAATGGCCCTTATGTTGATGAAC	-
PmamY_LHR-PFS fw	GTTCATCAACATAAGGGCCATTACTCGCATCCATTCTC	-
PmamY_PFS-RHR rev	CCTCACAAACAAAAGGCGGCGATTGAGGACCTTCAGTG	-
PmamY_PFS-RHR fw	CACTGAAGGTCCTCAATCGCCGCCTTTTGTTTGTGAGG	-
PmamY_RHR rev	CACTCCCTGCTGATTTCCTGGGAATAGCTC	-
PmamX_LHR fw	CGAACCATCCCACGCAGACGATGGAG	-
PmamX_LHR-PFS rev	GAATGGATGCGAGTAATGTGAACACCAAAGCCGTTG	-
PmamX_LHR-PFS fw	CAACGGCTTTGGTGTTCACATTACTCGCATCCATTC	-
PmamX_PFS-RHR rev	GTCCAGACGGAAGGCCGACGATTGAGGACCTTCAG	-
PmamX_PFS-RHR fw	CTGAAGGTCCTCAATCGTCGGCCTTCCGTCTGGAC	-
PmamX_RHR rev	GATCTTCATGCTCTCGCAGATTGTGAGAACCGTC	-
XhoI-Pmms6 fw	GAGCTCGAGACCTGTCAATTCCTTCCCTG	XhoI
BamH1-mmsF rev	CACGGATCCTCAGATCCGGTCGGCCAC	BamHI
PacI-Pmms36 fw	GGGTTAATTAAGGTTCGGCCTCGGGTGATTTAC	PacI
BamHI-mms48 rev	CACGGATCCTCATGTACTGCGGAACAGTCG	BamHI
XhoI-PmamH fw	GAGCTCGAGGTGTGCCACATTGCGAGG	XhoI
BamHI-mamH rev	CACGGATCCCTAGGCCACTTCGTCATC	BamHI
XhoI-PmamY fw	GGGCTCGAGATTATACTATTCCGAAATTAATAC	XhoI
SacI-mamY rev	GTTGAGCTCTCACGCATCGGAGATGG	SacI
XhoI-PmamX fw	GAACTCGAGCGAAAGCCGACGTCCGAAA	XhoI
SacI-ftsZ rev	GTTGAGCTCTCAGGCGATACCGGTGG	SacI
